# Development of a Platelet-Related Prognostic Model for Colorectal Cancer

**DOI:** 10.3389/fgene.2022.904168

**Published:** 2022-06-01

**Authors:** Pengcheng Wang, Wei Zhao, Hailei Cao

**Affiliations:** ^1^ Department of Colorectal and Anal Surgery, Shanxi Province Cancer Hospital, Taiyuan, China; ^2^ Department of Anesthesiology, First Affiliated Hospital of Harbin Medical University, Harbin, China

**Keywords:** colorectal cancer, platelet, subtype, prognostic model, tumor immunity

## Abstract

Colorectal cancer (CRC) represents one of the most common malignancies with high morbidity worldwide. Growing evidence has suggested that platelets are a fundamental component of the tumor microenvironment and play crucial roles in driving tumor biological behavior. The construction of a platelet-related prognostic model that can reliably predict CRC prognosis is of great clinical significance. The 1427 CRC-specific platelet-related genes were collected and mainly enriched in the ribosome and immune-related pathways. Based on platelet-related genes, three subtypes of TCGA CRC samples were identified by consensus clustering and characterized by differences in angiogenesis, epithelial–mesenchymal transition, immune infiltration, and prognosis. A total of 100 prognostic platelet-related genes were identified by univariate Cox regression. LASSO Cox regression further shrank those genes and constructed a 10-gene prognostic model. The patients with higher risk scores had significantly worse disease-specific survival than those with lower scores in both TCGA and validation cohorts. The risk score demonstrated good predictive performance for prognosis by receiver operating characteristic (ROC) curves. Furthermore, multivariate Cox regression analysis showed that the risk score was independent of TNM stage, sex, and age, and a graphic nomogram based on the risk score and clinical factors was developed to predict survival probability of CRC patients. Patients from the high-risk group were characterized by higher infiltration of immunosuppressive cells such as MDSC and Treg and higher expression of checkpoints *CTLA4*, *CD86*, and *PDCD1LG2*. Taken together, we identified three platelet-related subtypes and specifically constructed a promising 10-gene prognostic model in CRC. Our results highlighted the potential survival effects of platelet-related genes and provided evidence about their roles in regulating tumor immunity.

## Introduction

Colorectal cancer is the third most common cancer type and a leading cause of cancer-related mortality worldwide (Bray et al., 2018; Siegel et al., 20222022). The 5-year survival rate of CRC patients with stage I and II is 91 and 82%, respectively, while stage IV is approximately 12% (Miller et al., 20192019). The variation in survival of CRC patients due to tumor heterogeneity prompts an urgent need to develop new robust prognostic markers to improve predictive accuracy and complement the traditional TNM stage and tumor status. Moreover, a more effective risk stratification system is needed to aid in the management of CRC patients.

Growing evidence had showed that blood is a rich source of tumor-related biomarkers (Bardelli and Pantel, 2017; Kilgour et al., 2020), of which platelets are involved in a variety of tumor biological processes (Best et al., 2018), which can be used for biomarkers and diagnosis of cancer types (Best et al., 2018; Liu et al., 2020; Wurdinger et al., 2020). Platelet aggregation and degranulation along with the consequent release of platelet-derived proangiogenic mediators could influence tumor growth (Klinger and Jelkmann, 2002). Platelet-derived transforming growth factor-beta (TGF-β) and direct platelet–tumor cell interaction synergistically induce the epithelial–mesenchymal transition (EMT) in tumor cells and promote metastasis (Labelle et al., 2011; Miyashita et al., 2015). Furthermore, platelets can induce an immunosuppressive tumor microenvironment or support tumor cells to avoid immune elimination by protecting tumor cells directly from recognition by cytotoxic lymphocytes such as natural killer cells (McAllister and Weinberg, 2014; Best et al., 2018; Schmied et al., 2021).

Experimental data suggest that lower platelet count may reduce tumor growth and metastasis and could predict longer disease-specific survival (DFS) in diverse tumors (Møller Pedersen and Milman, 1996; Taucher et al., 2003; Ishizuka et al., 2012; Stone et al., 2012; Tao et al., 2021). In addition, platelet-related gene expression could be used to construct a prognostic model for predicting the survival of lung adenocarcinoma (Zhou et al., 2021). A recent study showed that the potential role of antiplatelet agents to suppress tumor progression by reducing platelet count or platelet activation was intriguing and validated. The platelet aggregation inhibitors exerted an inhibitory effect on metastatic spread (Kuznetsov et al., 2012), indicating that stratification of tumor samples based on the platelet-related biomarkers and antiplatelet agents therapy might be promising. A systematic study of tumor-educated blood platelets (TEPs) suggested that identified platelet-related genes enable CRC diagnostics (Best et al., 2015). However, whether these platelet-related genes are correlated with the prognosis in CRC patients remains to be studied.

Therefore, it is of great clinical significance to establish a platelet-related prognosis model to guide individual therapy. This study combined bioinformatics analysis and further validation on the basis of large biopsy samples to identify specific platelet-related subtypes and developed a reliable platelet-related prognostic risk scoring system in CRC.

## Materials and Methods

### CRC Sample Collection

The TCGA colorectal cancer (CRC) expression profile with log2 transformation was downloaded from the UCSC Xena browser (https://xenabrowser.net/), from which colon and rectal cancer samples were extracted. The clinical information of TCGA CRC samples was obtained from the cBioPortal website (http://www.cbioportal.org/) based on the R package “cgdsr”. The 324 CRC samples with available expression and disease-free survival (DFS) information were retained as the discovery cohort (Supplementary Table S1) and their corresponding characteristics were shown in Table 1.

**TABLE 1:** Clinical characteristics of patients in the TCGA cohort.

Characteristics	Variables	Number of samples
Age	Age <60	123
Age	Age >= 60	201
Lymphovascular invasion	Unknown	35
Lymphovascular. invasion	NO	201
Lymphovascular invasion	YES	88
Perineural invasion	Unknown	128
Perineural invasion	NO	146
Perineural invasion	YES	50
Sex	Female	142
Sex	Male	182
Stage	I/II	172
Stage	III/IV	138
Stage	Unknown	14
Tumor site	Unknown	2
Tumor site	Colon	245
Tumor site	Rectum	77

For a validation cohort, the gene expression data and the corresponding clinical information of 191 CRC samples were retrieved from the Gene Expression Omnibus database (GEO, https://www.ncbi.nlm.nih.gov/geo/) at accession number GSE161158. The corresponding characteristics of GSE161158 were detailed in Table 2.

**TABLE 2:** Clinical characteristics of patients in the GSE161158 cohort.

Characteristics	Variables	Number of samples
Age	Age <60	63
Age	Age >= 60	128
Stage	I/II	107
Stage	III/IV	84

### Functional Enrichment Analysis

The enrichment analysis of Gene Ontology (GO) and Kyoto Encyclopedia of Genes and Genomes (KEGG) was performed using the R package “clusterProfiler” with default parameters. We considered the *p*-value, adjusted by the BH method to control the FDR, less than 0.05 as the statistical significance and showed the top ten ones.

### Identification of Platelet-Related Subtypes

According to the previous study (Best et al., 2015), we obtained 2396 platelet-related genes in CRC and then screened out 1427 coding genes with |log2 fold change| (tumor patients vs. adjacent normal samples) greater than 1 for subsequent analysis (Supplementary Table S2). Based on the 1427 platelet-related genes, the R package “ConsensusClusterPlus” was applied to classify TCGA CRC samples into clusters (100 iterations, 80% resampling rate Pearson correlation). The best three-cluster separation was selected because the consensus clustering cumulative distribution function (CDF) curve in the range of 0.1–0.9 was near flat when the number of clusters *k* = 3.

### Gene Set Variation Analysis

To compare functional differences between platelet-related subtypes, we collected 15 angiogenesis and 11 EMT-relevant pathways from published resources including MSigDB, GO-BP, cancer hallmarks, REACTOME, and KEGG. Using gene set variation analysis (GSVA) from the R package “GSVA”, we calculated the activity score of the above pathways. Then the variation among subtypes was evaluated by the Wilcoxon rank-sum test. ns: not significant; ∗*p* < 0.05; ∗∗*p* < 0.01; ∗∗∗*p* < 0.001; ∗∗∗∗*p* < 0.0001.

### Estimation of Immune and Stromal Cell Infiltration

ESTIMATE (Yoshihara et al., 2013) was employed to infer the infiltrating extent of stromal and immune cells in tumor samples through the R package “estimate”. ESTIMATE is a popular algorithm, which was extensively utilized in tumor studies (Liu et al., 2022a; Liu et al., 2022b). To further explore the differences between immune cell subtypes, the CIBERSORT algorithm was used to assess the proportions of 22 immune cell subtypes based on the expression file (Newman et al., 2015). Based on a signature matrix of 547 genes, the 22 subtypes of infiltrating immune cells inferred by CIBERSORT include B cells, T cells, natural killer cells, macrophages, dendritic cells, eosinophils, and neutrophils. In addition, the gene signatures of 28 tumor infiltrating lymphocytes (TILs) were obtained from a previous study (Charoentong et al., 2017), and their activities in each sample were quantified using ssGSEA from the R package “GSVA”. The 28 subpopulations of TILs includes major types related to adaptive immunity: activated T cells, central memory (Tcm), effector memory (Tem) CD4^+^ and CD8^+^ T cells, gamma delta T (Tγδ) cells, T helper 1 (Th1) cells, Th2 cells, Th17 cells, regulatory T cells (Treg), follicular helper T cells (Tfh), activated, immature, and memory B cells, as well as cell types related to innate immunity, such as macrophages, monocytes, mast cells, eosinophils, neutrophils, activated, plasmacytoid, and immature dendritic cells (DCs), NK cells, natural killer T (NKT) cells, and MDSCs.

### Survival Analysis and Prognostic Model Construction

The 1427 candidate platelet-related genes in the TCGA CRC cohort were screened using univariate Cox regression analysis. Thus, 100 genes were identified as candidate biomarkers that were significantly related to DFS. Based on the “glmnet” in the R package, an optimal prognostic signature for CRC samples was built by LASSO Cox regression analysis using these candidate biomarkers. According to the optimal lambda value, a prognostic gene list with coefficients was generated from the LASSO model. Through multiplying the expression level of a prognostic gene by its corresponding LASSO coefficient, the risk score for each patient was calculated using the following formula: $\text{risk score}=\sum_{i=1}^{n}{\text{coef}}_{\text{i}}*{\text{expr}}_{\text{i}}$ , where coef_i_ is the LASSO coefficient of gene i, and expr_i_ is the expression level of the prognostic gene i. Then, the patients were separated into high- and low-risk groups based on the median value of the risk score. Kaplan–Meier survival curves with the log-rank tests were applied to compare differences in survival between the patient groups. And a time-dependent ROC curve analysis was used to evaluate the predictive efficacy using the R package “timeROC”.

### Statistical Analysis

The Wilcoxon rank-sum test was conducted to compare the continuous variables between the two groups, such as gene expression level, immune infiltration, pathway activity, and risk score. The univariate Cox regression analyses and LASSO regression analyses were applied to identify the most significant platelet-related genes. The multivariate Cox regression analyses were used to confirm the risk score as an independent prognostic factor after adjusting for clinical factors. Kaplan–Meier analysis and the log-rank test were employed to evaluate correlations between groups and DFS. ROC was used to study the prediction efficiency of the prognostic model. The prognostic value of the nomogram was evaluated by the C-index. In the present study, all tests of significance were two-sided, and *p* < 0.05 was considered statistically significant. ns: not significant; ∗*p* < 0.05; ∗∗*p* < 0.01; ∗∗∗*p* < 0.001; ∗∗∗∗*p* < 0.0001.

## Results

### Function Enrichment Analysis of Platelet-Related Genes

**FIGURE 1:**
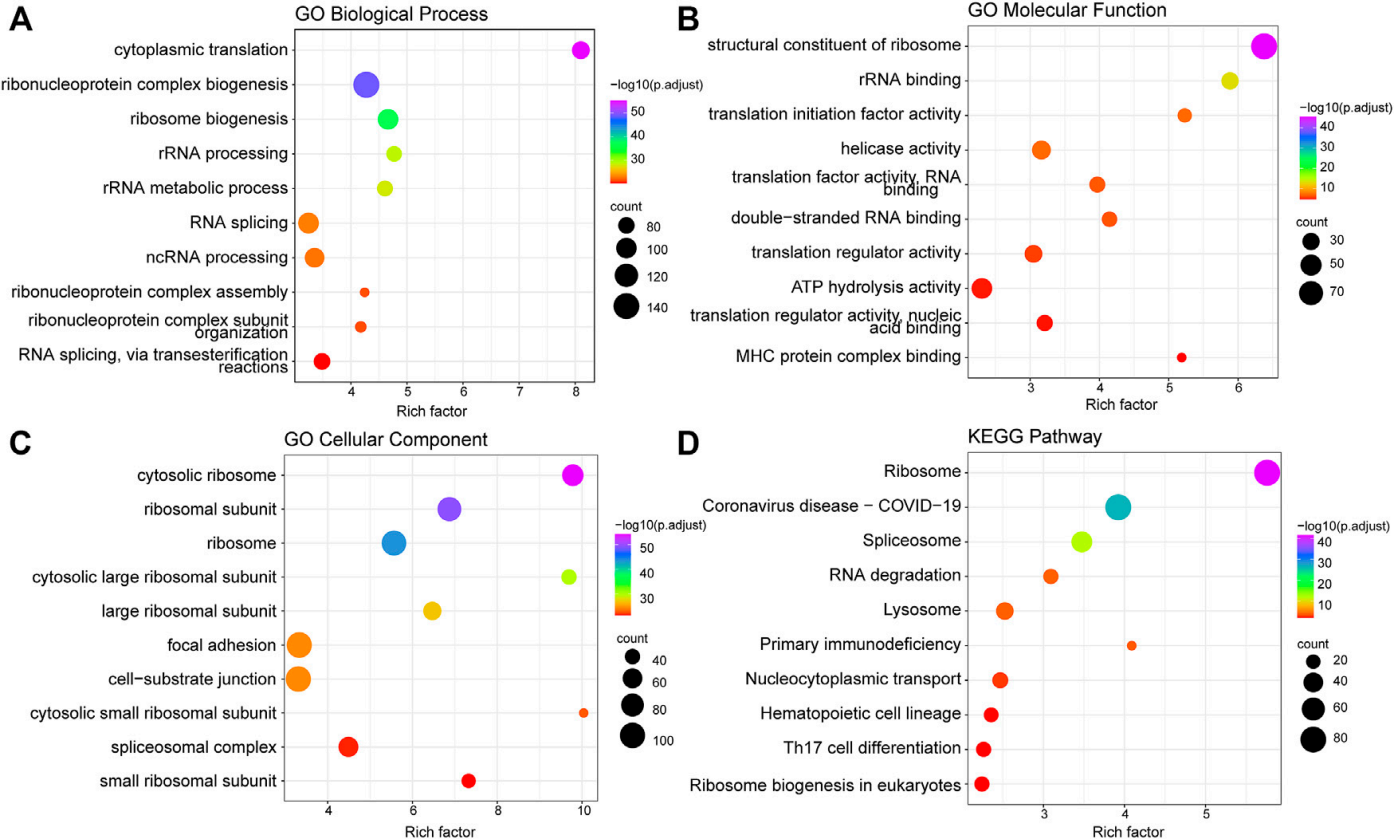
Function enrichment analysis of platelet-related genes. **(A)** Top 10 significantly enriched biological processes of GO. **(B)** Top 10 significantly enriched molecular functions of GO. **(C)** Top 10 significantly enriched cell components of GO. **(D)** Top 10 significantly enriched KEGG pathways. The size of the points represents the number of genes annotated to the pathway, and the color represents significance.

After screening out 1427 candidate platelet-related genes, GO terms and KEGG pathway analyses were performed to further investigate their involvement in the biological process (for details, see Methods). GO analysis revealed that platelet-related genes were mainly enriched in cytoplasmic translation, ribosomal processing, and ribosomal metabolism biological processes (Figure 1A). This analysis additionally showed that these genes were chiefly involved in translation factors activity, ribosomal binding, and MHC protein complex binding under the molecular function category (Figure 1B). Furthermore, the enriched cellular components included cytosolic ribosome, cytosolic large ribosomal subunit, and cytosolic small ribosomal subunit (Figure 1C).

In regards to the KEGG pathway analysis, platelet-related genes were also significantly enriched in ribosome-related pathways (Figure 1D), which was consistent with the previous findings that protein synthesis continues in platelets through dynamic regulation of a ribosome rescue pathway, even though platelets lack nuclei to produce new mRNA and ribosomes (Ji et al., 2011; Mills et al., 2016). In addition, the major involvement in some immune-related pathways was also shown in KEGG results, such as primary immune deficiency, hematopoietic cell regulation, and Th17 cell differentiation (Figure 1D).

### Identification and Characterization of Platelet-Related Subtypes in Colorectal Cancer

**FIGURE 2:**
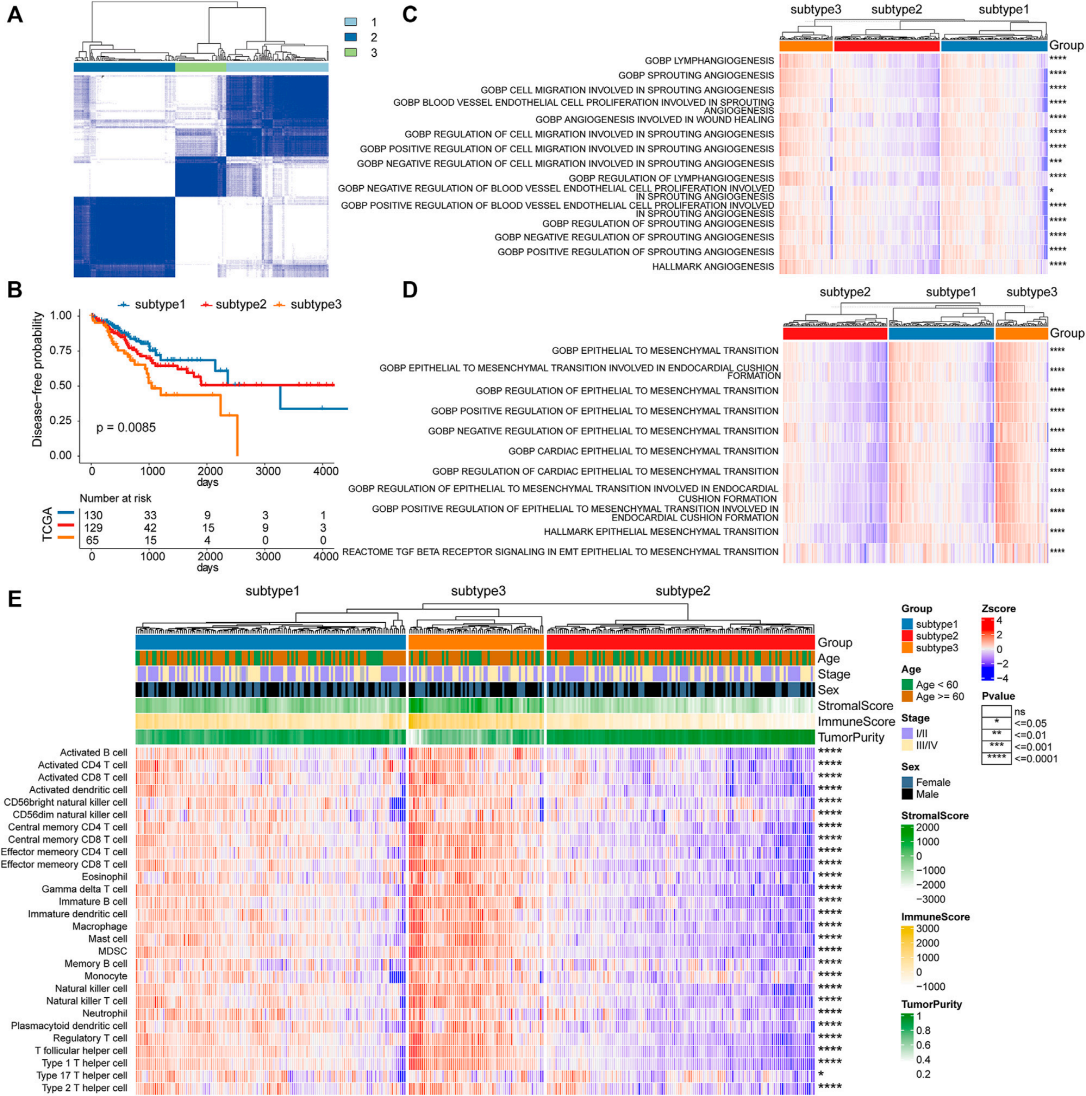
Platelet-related subtypes in CRC. **(A)** Consensus clustering of the pairwise correlation between the CRC samples (rows and columns) in the TCGA cohort based on the expression of platelet-related genes. **(B)** Kaplan–Meier curves for DFS by three platelet-related subtypes. **(C)** The activity score of 15 angiogenesis pathways between three platelet-related subtypes, as well as 11 EMT-related pathways shown in **(D)**. **(E)** The infiltration of 28 immune cell subtypes between three platelet-related subtypes. The features (rows of heatmap) between subtype3 and the other two subtypes were compared based on the Wilcoxon rank-sum test, where significance was labeled in the right of each row.

Based on the platelet-related genes, unsupervised consensus clustering distinguished the 324 TCGA CRC tumors into three distinct groups termed platelet-related subtype1 (*n* = 130), subtype2 (*n* = 129), and subtype3 (*n* = 65) respectively (details see Methods; Figure 2A). Further exploration revealed significant differences in survival between three subtypes (*p* = 0.0085), among which the subtype3 had the worst DFS (Figure 2B). The mechanisms by which platelets contribute to tumor growth and metastasis include their potential to promote angiogenesis, EMT, and immune evasion (Best et al., 2018). Therefore, we further explored whether there are differences in those biological processes among the three platelet-related subtypes (details see Methods; Supplementary Table S3). The activity score of 15 angiogenesis and 11 EMT-related pathways were consistently significantly higher in subtype3 than in the other two subtypes, suggesting that subtype3 has the highest stromal activity and greatest ability to promote tumor progression (Figures 2C,D).

Since platelet-related genes were found to be involved in regulating immune-related pathways (Figure 1D), we further explored differences in immune microenvironment among distinct subtypes. To our surprise, the proportion of innate immune cell infiltration in subtype3 was also significantly higher than that in the other two subtypes, such as natural killer cells, macrophages, eosinophils, and mast cells (Figure 2E). Previous studies have shown that tumors with an immune exclusion phenotype also show the presence of large numbers of immune cells but remain in the stroma around the tumor cell nest rather than penetrating its parenchyma (Zhang et al., 2020). Additionally, we analyzed the oncogenic features of the three subtypes in CRC and found that subtype3 showed the highest oncogenic activities (Supplementary Figure S1), including cancer-associated fibroblasts (CAFs), angiogenesis, and tumor-associated macrophages (TAM). Previous studies have shown that CAFs play a crucial role in the development of desmoplastic reactions and shape the tumor immune microenvironment in CRC (Koliaraki et al., 2017; Mochizuki et al., 2020). CAFs may, directly and indirectly, impact anti-tumor immune reaction through the recruitment of protumorigenic inflammatory cells, such as M2-like TAM (Zhang and Liu, 2013).

To validate the performance of subtypes, we conducted cluster analysis in the validation cohort (GSE161158, Supplementary Figure S2A). The results showed that there was a consistent phenomenon between the TCGA CRC cohort and the validation cohort. There were significant differences in patient survival among the three subtypes in the validation cohort (Supplementary Figure S2B). In addition, in line with the TCGA CRC cohort, the infiltration of 28 immune cells was obviously different among the three subtypes in the validation cohort (Supplementary Figure S2C). In general, platelet-related subtypes were characterized by differences in angiogenesis, epithelial-mesenchymal transition, immune infiltration, and prognosis, indicating the potential of platelet-related genes to predict prognosis in CRC.

### Identification of Prognostic Platelet-Related Genes and Construction of a Prognostic Model

**FIGURE 3:**
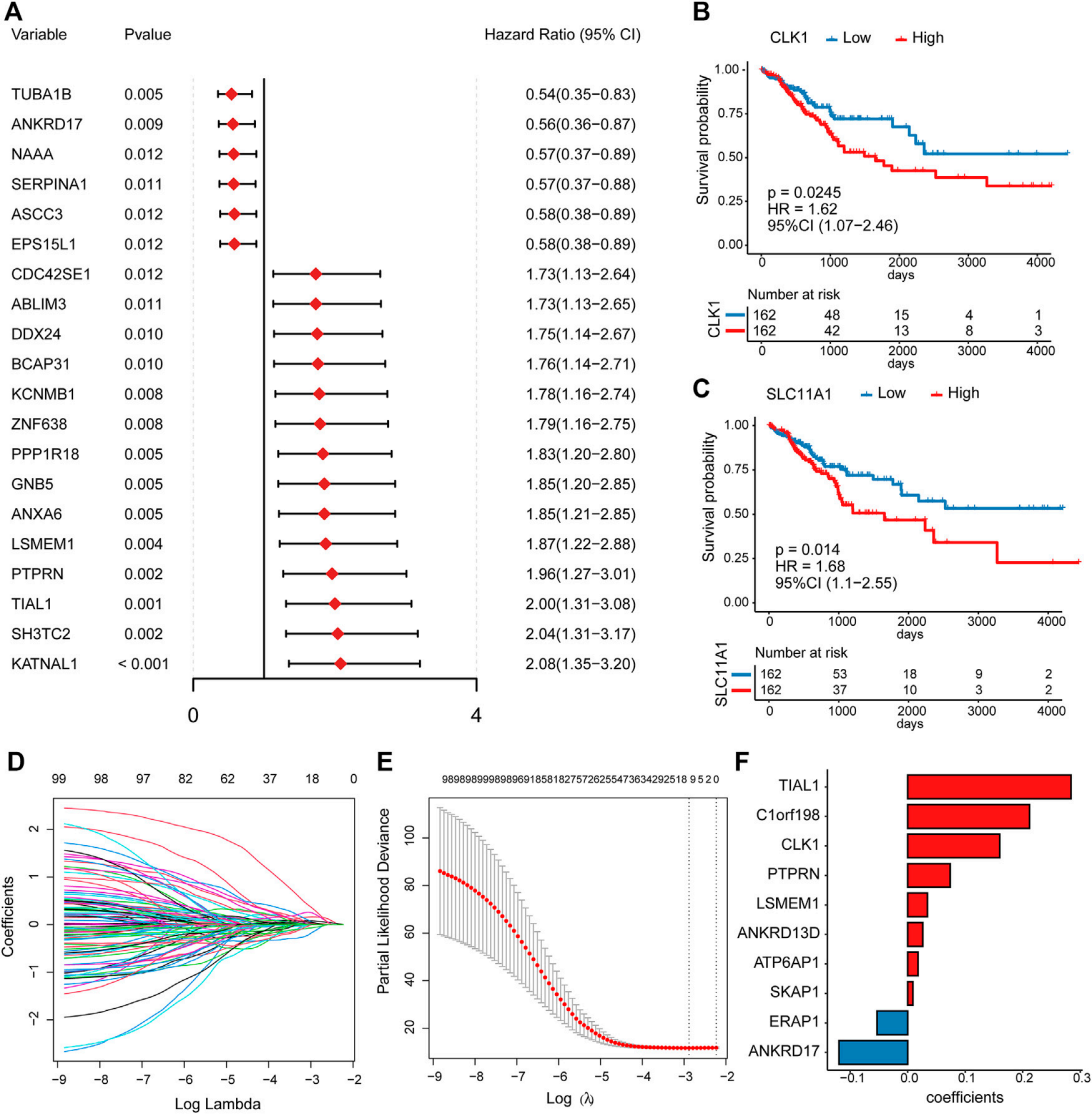
The univariate Cox regression and LASSO Cox analysis. **(A)** The forest plot for hazard ratio and *p*-value of the top 20 prognostic platelet-related genes from the univariable Cox regression in the TCGA cohort. **(B,C)** Kaplan–Meier curves for DFS by expression of *CLK1* as well as *SLC11A1*, where the median expression was cut-off. **(D)** LASSO coefficient profiles of the prognostic genes. **(E)** The partial likelihood deviance plot presented the minimum number corresponding to the covariates used for LASSO Cox analysis. **(F)** LASSO coefficients of the 10 predictor genes for constructing the prognostic model.

To better investigate the prognostic role of platelet-related genes in CRC, univariate Cox regression analysis was applied in the TCGA cohort. The results showed that 100 genes were significantly associated with the DFS out of 1427 platelet-related genes (Supplementary Table S4). The top 20 prognostic platelet-related genes were shown in Figure 3A. Some of these genes had already been reported as prognostic biomarkers, such as *CLK1* (*p* = 0.0245) (Yang et al., 2021) and *SLC11A1* (*p* = 0.014) (Zhu et al., 2022) (Figures 3B,C).

To remove redundant prognostic factors, we performed a LASSO Cox analysis on 100 prognostic platelet-related genes. Ten predictors with the greatest influence on DFS in CRC patients were determined (Supplementary Table S5), of which 8 were risk genes (*TIAL1*, *C1orf198*, *CLK1*, *PTPRN*, *LSMEM1*, *ANKRD13D*, *ATP6AP1*, and *SKAP1*), and 2 were protective genes (*ERAP1* and *ANKRD17*) (Figures 3D–F). Previous studies supported that *CLK1* and *ANKRD17* could be served as biomarkers or potential therapeutic targets for CRC (Ioana et al., 2010; Lian et al., 2020). Then, these 10 predictors were subjected to construct a platelet-related prognostic risk score using a linear combination of each gene expression level and its risk coefficient Figure 3E (details see Methods; Supplementary Table S5).

### The Prognostic Capacity of the Risk Score

**FIGURE 4:**
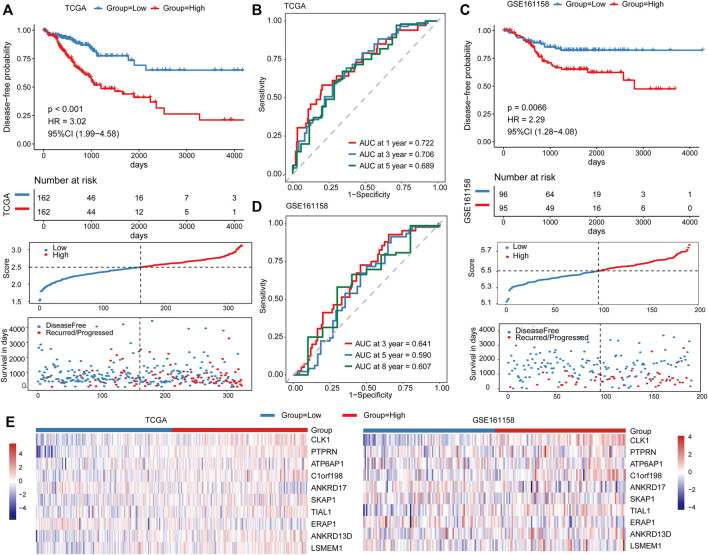
Prognostic performance of the risk score in the TCGA and validation cohorts. Kaplan–Meier curves for DFS by risk groups (top), distributions of risk scores (middle), and survival statuses of patients between low‐risk and high‐risk groups (bottom) in the TCGA cohort **(A)** and validation cohort **(C)**. ROC analysis of the risk score in the TCGA cohort **(B)** and validation cohort **(D)**. **(E)** The expression distribution of 10 predictor genes in patients from the TCGA cohort (left) and validation cohort (right).

Risk scores were calculated based on a 10-gene prognostic model. Patients in the training dataset TCGA cohort were then stratified into high-risk group (*n* = 162) and low-risk group (*n* = 162) based on the median risk score. The Kaplan–Meier survival analysis confirmed the high-risk group yielding reduced survival time (*p* < 0.001, Figure 4A). The predictive performance of the prognostic model was evaluated by the time-dependent ROC curves and the area under the curve (AUC) reached 0.722 at 1 year, 0.706 at 3 years, and 0.689 at 5 years, suggesting that this prognostic model exhibited good sensitivity and specificity (Figure 4B). In addition, we assessed the prognostic efficacy of risk score for all available patient information and found that risk score was associated with poor prognosis under different clinical factors (Supplementary Figure S3). Furthermore, we calculated the concordance index (C-index) of the model under the conditions of risk score, clinical factors, and risk score and clinical factors, respectively. The results showed that the prognostic efficacy of combined status (C-index = 0.76) was better than that of risk score alone (C-index = 0.61) and clinical factors alone (C-index = 0.65, Supplementary Figure S4A).

We compared the prognosis performance of the risk score in this study with the genes that were associated with CRC prognosis (including *CLK1* and *SLC11A1*). Our results revealed that the risk score exceeded CRC prognosis both in C-index and AUC value at different time points, which indicated that the prognosis performance of our signature was better than CRC prognosis (Supplementary Figures S5A,B). To test the robustness of the model, the risk score was calculated for 190 patients as the independent validation cohort (GSE161158). Similarly, patients in the high-risk group exhibited markedly poorer survival than those in the low-risk group (*p* < 0.001, Figure 4C). The AUCs for 3, 5, and 8 years were 0.641, 0.590, and 0.607, respectively (Figure 4D). In addition, the expression distributions of these 10 prognostic predictor genes in the TCGA and validation cohorts were consistent between the high- and low-risk groups (Figure 4E).

### Clinicopathological Significance and Independence of the Risk Score

**FIGURE 5:**
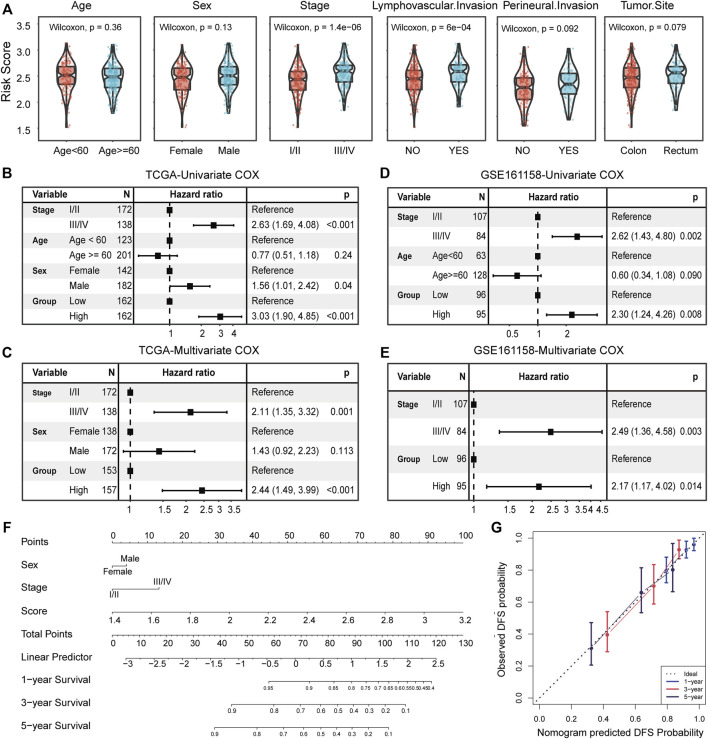
Clinicopathological association and independence of the risk score. **(A)** Comparison of the risk score between patient groups by clinicopathological features, including age, sex, TNM stage, lymphatic vascular invasion, perineural invasion, and tumor anatomic site. Results of the univariate and multivariate Cox regression analyses for the risk score and clinicopathological features in the TCGA cohort **(B,C)** and independent validation cohort **(D,E)**. **(F)** Nomogram based on the TNM stage, sex, and risk score for 1-, 3-, and 5-year DFS prediction. **(G)** Calibration plot for agreement test between 1-, 3-, and 5-year DFS prediction and actual observation.

Subsequently, we further investigate the associations between risk scores and clinicopathological features. The risk scores of patients with the advanced stage was significantly higher than that of patients with early stage (*p* = 1.4e-06), and that of patients with lymphatic vascular invasion was higher than that of patients without invasion (*p* = 6e-04, Wilcoxon rank-sum test; Figure 5A). No other significant correlations were found between risk score and clinicopathological features, such as age, sex, perineural invasion, and tumor anatomic site.

Univariate and multivariate Cox regression analyses were conducted to assess the independent predictive power of risk score. In the TCGA cohort, the univariate analysis indicated that TNM stage (I/II or III/IV), sex (female or male), and risk score (high or low) were significantly correlated with DFS (*p* < 0.05; Figure 5B), especially for risk score (HR = 3.03, 95% CI 1.90–4.85, *p* < 0.001; Figure 5B). After adjusting for clinical factors, the risk score remained a significant independent prognostic factor (HR = 2.44, 95% CI 1.49–3.99, *p* < 0.001; Figure 5C). We extra reconsidered patient information (including lymphovascular invasion, perineural invasion, venous invasion, and tumor site), and found that when we corrected these clinical factors, our risk score remained a significant independent prognostic factor (Supplementary Figure S4B). Correspondingly, the independent prognostic value of risk scores was also confirmed in the validation cohort (GSE161158) (*p* = 0.014; Figures 5D,E). In addition, a graphic nomogram based on the TNM stage, sex, and risk score was developed to predict the 1-, 3-, and 5-year survival probability of TCGA CRC patients (Figure 5F).

The calibration plot showed an optimal agreement between the prediction by the nomogram and actual observations (Figure 5G), indicating the potential of the risk score to construct a combination marker. Overall, the risk score not only serves as a new independent and robust prognostic marker but also complements the traditional TNM stage.

### Comparison of Immune-Related Features Between Different Risk Groups

**FIGURE 6:**
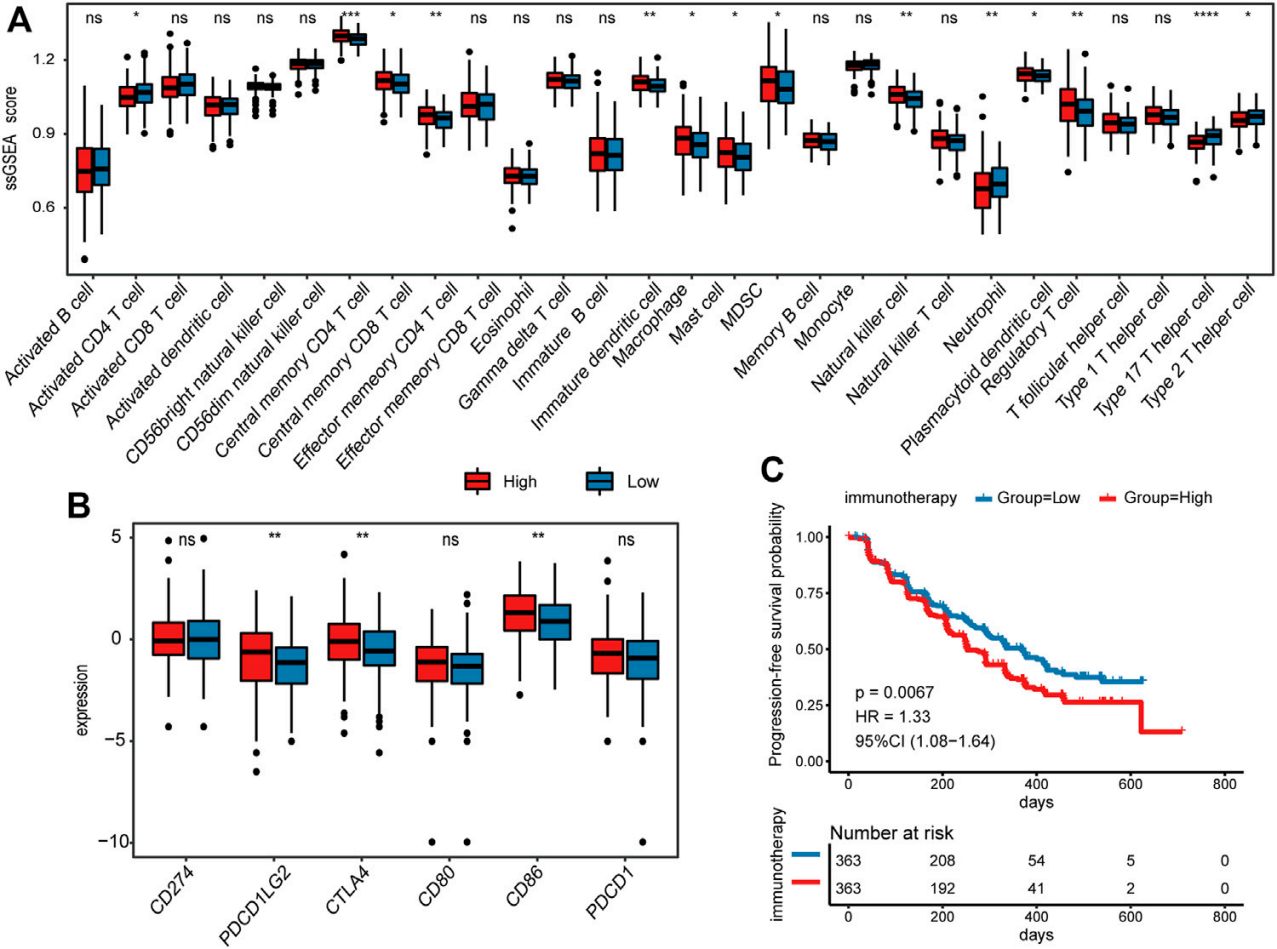
Comparison of immune-related features between high- and low-risk groups. **(A)** Comparison of the infiltration of 28 immune cell types between high- and low-risk groups in TCGA CRC cohort. **(B)** Comparison of 6 immune checkpoints expression between high- and low-risk groups in TCGA CRC cohort. **(C)** Kaplan–Meier survival analysis for PFS in immunotherapy cohort.

To further explore the differences in immune status between high- and low-risk groups, we calculated the infiltration of 28 immune cell subtypes by ssGSEA (details see Methods). The infiltration of macrophage, MDSC, and Treg were significantly increased in the high-risk group of the TCGA CRC cohort (Wilcoxon rank-sum test, *p* < 0.05; Figure 6A), which was consistent with their role in facilitating tumor growth, angiogenesis, invasion, as well as metastasis (Goswami et al., 2017; Hashemi Goradel et al., 2019; Olguín et al., 2020). Moreover, immature dendritic cells, memory CD8 T cell, and memory CD4 T-cell subtypes were also increased in the high-risk group. On the contrary, infiltrations of activated CD4 T cells, neutrophils, Th17 cells, and Type 2 T helper cells were significantly reduced (Wilcoxon rank-sum test, *p* < 0.05; Figure 6A). Subsequently, the immune infiltration assessed by the CIBERSORT algorithm supported the above results (Supplementary Table S6).

In addition to immune infiltration, we also investigated the immune checkpoint molecules expressed by immune cells that regulate anti-tumor immune responses. Among the 6 immune checkpoints, *CTLA4*, *CD86*, and *PDCD1LG2* were highly expressed in the high-risk group (Wilcoxon rank-sum test, *p* < 0.05; Figure 6B), suggesting that these genes may serve as key targets for anti-tumor therapy in CRC. In addition, we applied the prognostic model to 726 patients who received immunotherapy (Killock, 2020), and divided them into low- and high-risk groups. Interestingly, the results showed that high-risk scores indicated worse progression-free survival (PFS) (*p* = 0.0067; Figure 6C), implying the potential of platelet-related genes as prognostic markers for immunotherapy.

## Discussion

CRC is characterized by a high socioeconomic burden, high morbidity, and mortality (Bray et al., 2018). However, effective prognostic biomarkers in CRC are urgently needed. Accumulated evidence demonstrated that tumor-educated platelets played an important role in tumor progression (Joosse and Pantel, 2015; Best et al., 2018). Therefore, by means of the prominent effectiveness of transcriptome analysis on clinical issues (Cieslik and Chinnaiyan, 2018; Rodon et al., 2019; Sailer et al., 2019), we explored the value of platelet-related genes in the clinical application of CRC, with particular attention to prognosis. In this study, we identified three platelet-related subtypes and specifically constructed a 10-gene prognostic model to predict DFS in CRC. Among them, many genes were discovered to have the potential to predict prognosis in CRC. For example, *TIAL1* was top significant in both univariate and LASSO Cox regression analysis and was found to be a prognostic risk factor for breast cancer (Aritake et al., 2004; Suvanto et al., 2020). However, the relationship between *TIAL1* with CRC is rarely reported. These findings provide a new perspective on CRC prognosis based on platelet-related genes. Interestingly, the risk score calculated by our prognostic model was independent of the TNM stage in multivariate Cox analyses, although it was significantly higher in advanced tumors. Moreover, the risk score was demonstrated as a complement to the traditional TNM staging by the nomograms analysis. These results suggested that the risk score may serve as a transcriptomic predictor of CRC metastasis and aid in the development of liquid blood markers for CRC in the future.

We found that platelet-related subtype 3 had significantly higher activities of angiogenesis and EMT pathway, which have been confirmed as hallmarks of cancer (Hanahan and Weinberg, 2011). Importantly, subtype 3 did have the worst survival. In addition, both subtype3 and high-risk groups had significantly higher infiltration of macrophages and NK cells. Macrophages promoted tumor progression (Goswami et al., 2017) and platelets protected tumors from NK cells with killing capacity (Schlesinger, 2018), which was consistent with previous studies. Platelets are the major source of TGF-β in the tumor microenvironment by expression of the TGF-β-docking receptor GARP, which is highly expressed by activated Tregs (Wallace et al., 2018; Kopp et al., 2009). In this study, we found that the patients with subtype3 had significantly higher levels of TGFβ activities (Z-Score) and Tregs infiltration than other patients (Supplementary Figures S6A,B). A previous study revealed that the GARP-TGFβ complex together with platelet-secreted lactate inhibited T-cell immunity against both melanoma and colon cancer (Rachidi et al., 2017; Metelli et al., 2018), which supports our study (Supplementary Figures S6A,B).

Previous studies have shown that Th17 cells could promote cytotoxic T-cell activation in tumor immunity, and reduce tumor growth (Martin-Orozco et al., 2009; Canderan and Dellabona, 2010). In our study, Th17 infiltration was significantly higher in the low-risk group of CRC patients with a good prognosis, which is in line with previous studies. The immune checkpoints *CTLA4*, *CD86*, and *PDCD1LG2* were found highly expressed in the high-risk group. Together, these findings suggested that platelet-related subtypes or risk groups have intricate mechanisms that contribute to tumor progression and thus affect survival in patients with CRC. In this study, some shortcomings and prospects should be addressed. This study was completely based on public databases, and some key genes or results need to be externally validated by further experiments. For example, the biological functions of the 10 predictor genes, the relationship between risk scores, and the clinical benefits of antiplatelet agents in CRC need to be verified in the future.

In conclusion, our study firstly revealed a significant association between platelet-related genes and prognosis in CRC. Three platelet-related subtypes were identified and a platelet-related prognostic model was constructed as an independent prognostic risk predictor in CRC. Validation of the external dataset further confirmed the independence and predictive performance of this model. This study deepens our understanding of platelet-related genes in CRC and provides new potential prognostic and therapeutic biomarkers.

## Data Availability

The original contributions presented in the study are included in the article/Supplementary Material; further inquiries can be directed to the corresponding author.

## References

[B1] Aritake N. Tamaki Y. Masuda N. Nakano Y. Monden T. Noguchi S. (2004). High Expression of Two Genes Selected by iAFLP: a New Prognostic Factor of Estrogen Receptor-Positive Breast Cancer. Oncol. Rep. 12 (2), 381–387. 10.3892/or.12.2.381 15254706

[B2] Bardelli A. Pantel K. (2017). Liquid Biopsies, what We Do Not Know (Yet). Cancer Cell. 31 (2), 172–179. 10.1016/j.ccell.2017.01.002 28196593

[B3] Best M. G. Wesseling P. Wurdinger T. (2018). Tumor-Educated Platelets as a Noninvasive Biomarker Source for Cancer Detection and Progression Monitoring. Cancer Res. 78 (13), 3407–3412. 10.1158/0008-5472.CAN-18-0887 29921699

[B4] Best M. G. Sol N. Kooi I. Tannous J. Westerman B. A. Rustenburg F. (2015). RNA-seq of Tumor-Educated Platelets Enables Blood-Based Pan-Cancer, Multiclass, and Molecular Pathway Cancer Diagnostics. Cancer Cell. 28 (5), 666–676. 10.1016/j.ccell.2015.09.018 26525104 PMC4644263

[B5] Bray F. Ferlay J. Soerjomataram I. Siegel R. L. Torre L. A. Jemal A. (2018). Global Cancer Statistics 2018: GLOBOCAN Estimates of Incidence and Mortality Worldwide for 36 Cancers in 185 Countries. CA A Cancer J. Clin. 68 (6), 394–424. 10.3322/caac.21492 30207593

[B6] Canderan G. Dellabona P. (2010). T Helper 17 T Cells Do Good for Cancer Immunotherapy. Immunotherapy 2 (1), 21–24. 10.2217/imt.09.83 20635888

[B7] Charoentong P. Finotello F. Angelova M. Mayer C. Efremova M. Rieder D. (2017). Pan-cancer Immunogenomic Analyses Reveal Genotype-Immunophenotype Relationships and Predictors of Response to Checkpoint Blockade. Cell. Rep. 18 (1), 248–262. 10.1016/j.celrep.2016.12.019 28052254

[B8] Cieslik M. Chinnaiyan A. M. (2018). Cancer Transcriptome Profiling at the Juncture of Clinical Translation. Nat. Rev. Genet. 19 (2), 93–109. 29279605 10.1038/nrg.2017.96

[B9] Goswami K. K. Ghosh T. Ghosh S. Sarkar M. Bose A. Baral R. (2017). Tumor Promoting Role of Anti-tumor Macrophages in Tumor Microenvironment. Cell. Immunol. 316, 1–10. 10.1016/j.cellimm.2017.04.005 28433198

[B10] Hanahan D. Weinberg R. A. (2011). Hallmarks of Cancer: the Next Generation. Cell. 144 (5), 646–674. 10.1016/j.cell.2011.02.013 21376230

[B11] Hashemi Goradel N. Heidarzadeh S. Jahangiri S. Farhood B. Mortezaee K. Khanlarkhani N. (2019). Fusobacterium Nucleatum and Colorectal Cancer: A Mechanistic Overview. J. Cell. Physiology 234 (3), 2337–2344. 10.1002/jcp.27250 30191984

[B12] Ioana M. Angelescu C. Burada F. Mixich F. Riza A. Dumitrescu T. (2010). MMR Gene Expression Pattern in Sporadic Colorectal Cancer. J. Gastrointestin Liver Dis. 19 (2), 155–159. 20593048

[B13] Ishizuka M. Nagata H. Takagi K. Iwasaki Y. Kubota K. (2012). Preoperative Thrombocytosis Is Associated with Survival after Surgery for Colorectal Cancer. J. Surg. Oncol. 106 (7), 887–891. 10.1002/jso.23163 22623286

[B14] Ji P. Murata-Hori M. Lodish H. F. (2011). Formation of Mammalian Erythrocytes: Chromatin Condensation and Enucleation. Trends Cell. Biol. 21 (7), 409–415. 10.1016/j.tcb.2011.04.003 21592797 PMC3134284

[B15] Joosse S. A. Pantel K. (2015). Tumor-Educated Platelets as Liquid Biopsy in Cancer Patients. Cancer Cell. 28 (5), 552–554. 10.1016/j.ccell.2015.10.007 26555171

[B16] Kilgour E. Rothwell D. G. Brady G. Dive C. (2020). Liquid Biopsy-Based Biomarkers of Treatment Response and Resistance. Cancer Cell. 37 (4), 485–495. 10.1016/j.ccell.2020.03.012 32289272

[B17] Killock D. (2020). Biomarkers 101 - Personalizing Therapy for RCC. Nat. Rev. Clin. Oncol. 17 (11), 653. 10.1038/s41571-020-00434-4 32948859

[B18] Klinger M. H. F. Jelkmann W. (2002). Review: Role of Blood Platelets in Infection and Inflammation. J. Interferon & Cytokine Res. 22 (9), 913–922. 10.1089/10799900260286623 12396713

[B19] Koliaraki V. Pallangyo C. K. Greten F. R. Kollias G. (2017). Mesenchymal Cells in Colon Cancer. Gastroenterology 152 (5), 964–979. 10.1053/j.gastro.2016.11.049 28111227

[B20] Kopp H.-G. Placke T. Salih H. R. (2009). Platelet-Derived Transforming Growth Factor-β Down-Regulates NKG2D Thereby Inhibiting Natural Killer Cell Antitumor Reactivity. Cancer Res. 69 (19), 7775–7783. 10.1158/0008-5472.can-09-2123 19738039

[B21] Kuznetsov H. S. Marsh T. Markens B. A. Castaño Z. Greene-Colozzi A. Hay S. A. (2012). Identification of Luminal Breast Cancers that Establish a Tumor-Supportive Macroenvironment Defined by Proangiogenic Platelets and Bone Marrow-Derived Cells. Cancer Discov. 2 (12), 1150–1165. 10.1158/2159-8290.cd-12-0216 22896036 PMC3517696

[B22] Labelle M. Begum S. Hynes R. O. (2011). Direct Signaling between Platelets and Cancer Cells Induces an Epithelial-mesenchymal-like Transition and Promotes Metastasis. Cancer Cell. 20 (5), 576–590. 10.1016/j.ccr.2011.09.009 22094253 PMC3487108

[B23] Lian H. Wang A. Shen Y. Wang Q. Zhou Z. Zhang R. (2020). Identification of Novel Alternative Splicing Isoform Biomarkers and Their Association with Overall Survival in Colorectal Cancer. BMC Gastroenterol. 20 (1), 171. 10.1186/s12876-020-01288-x 32503434 PMC7275609

[B24] Liu L. Lin F. Ma X. Chen Z. Yu J. (2020). Tumor-educated Platelet as Liquid Biopsy in Lung Cancer Patients. Crit. Rev. Oncology/Hematology 146, 102863. 10.1016/j.critrevonc.2020.102863 31935617

[B25] Liu Z. Liu L. Weng S. Guo C. Dang Q. Xu H. (2022). Machine Learning-Based Integration Develops an Immune-Derived lncRNA Signature for Improving Outcomes in Colorectal Cancer. Nat. Commun. 13 (1), 816. 10.1038/s41467-022-28421-6 35145098 PMC8831564

[B26] Liu Z. Xu H. Weng S. Ren Y. Han X. (2022). Stemness Refines the Classification of Colorectal Cancer with Stratified Prognosis, Multi-Omics Landscape, Potential Mechanisms, and Treatment Options. Front. Immunol. 13, 828330. 10.3389/fimmu.2022.828330 35154148 PMC8828967

[B27] Martin-Orozco N. Muranski P. Chung Y. Yang X. O. Yamazaki T. Lu S. (2009). T Helper 17 Cells Promote Cytotoxic T Cell Activation in Tumor Immunity. Immunity 31 (5), 787–798. 10.1016/j.immuni.2009.09.014 19879162 PMC2787786

[B28] McAllister S. S. Weinberg R. A. (2014). The Tumour-Induced Systemic Environment as a Critical Regulator of Cancer Progression and Metastasis. Nat. Cell. Biol. 16 (8), 717–727. 10.1038/ncb3015 25082194 PMC6220424

[B29] Metelli A. Salem M. Wallace C. H. Wu B. X. Li A. Li X. (2018). Immunoregulatory Functions and the Therapeutic Implications of GARP-TGF-β in Inflammation and Cancer. J. Hematol. Oncol. 11 (1), 24. 10.1186/s13045-018-0570-z 29458436 PMC5819195

[B30] Miller K. D. Nogueira L. Mariotto A. B. Rowland J. H. Yabroff K. R. Alfano C. M. (2019). Cancer Treatment and Survivorship Statistics, 2019. CA A Cancer J. Clin. 69 (5), 363–385. 10.3322/caac.21565 31184787

[B31] Mills E. W. Wangen J. Green R. Ingolia N. T. (2016). Dynamic Regulation of a Ribosome Rescue Pathway in Erythroid Cells and Platelets. Cell. Rep. 17 (1), 1–10. 10.1016/j.celrep.2016.08.088 27681415 PMC5111367

[B32] Miyashita T. Tajima H. Makino I. Nakagawara H. Kitagawa H. Fushida S. (2015). Metastasis-promoting Role of Extravasated Platelet Activation in Tumor. J. Surg. Res. 193 (1), 289–294. 10.1016/j.jss.2014.07.037 25173834

[B33] Mochizuki S. Ao T. Sugiura T. Yonemura K. Shiraishi T. Kajiwara Y. (2020). Expression and Function of a Disintegrin and Metalloproteinases in Cancer-Associated Fibroblasts of Colorectal Cancer. Digestion 101 (1), 18–24. 10.1159/000504087 31722362

[B34] Møller Pedersen L. Milman N. (1996). Prognostic Significance of Thrombocytosis in Patients with Primary Lung Cancer. Eur. Respir. J. 9 (9), 1826–1830. 10.1183/09031936.96.09091826 8880098

[B35] Newman A. M. Liu C. L. Green M. R. Gentles A. J. Feng W. Xu Y. (2015). Robust Enumeration of Cell Subsets from Tissue Expression Profiles. Nat. Methods 12 (5), 453–457. 10.1038/nmeth.3337 25822800 PMC4739640

[B36] Olguín J. E. Medina-Andrade I. Rodríguez T. Rodríguez-Sosa M. Terrazas L. I. (2020). Relevance of Regulatory T Cells during Colorectal Cancer Development. Cancers (Basel) 12 (7). 10.3390/cancers12071888 PMC7409056 32674255

[B37] Rachidi S. Metelli A. Riesenberg B. Wu B. X. Nelson M. H. Wallace C. (2017). Platelets Subvert T Cell Immunity against Cancer *via* GARP-Tgfβ axis. Sci. Immunol. 2 (11), eaai7911. 10.1126/sciimmunol.aai7911 28763790 PMC5539882

[B38] Rodon J. Soria J.-C. Berger R. Miller W. H. Rubin E. Kugel A. (2019). Genomic and Transcriptomic Profiling Expands Precision Cancer Medicine: the WINTHER Trial. Nat. Med. 25 (5), 751–758. 10.1038/s41591-019-0424-4 31011205 PMC6599610

[B39] Sailer V. Eng K. W. Zhang T. Bareja R. Pisapia D. J. Sigaras A. (2019). Integrative Molecular Analysis of Patients with Advanced and Metastatic Cancer. JCO Precis. Oncol. 3, PO.19.00047. 10.1200/PO.19.00047 PMC6778956 31592503

[B40] Schlesinger M. (2018). Role of Platelets and Platelet Receptors in Cancer Metastasis. J. Hematol. Oncol. 11 (1), 125. 10.1186/s13045-018-0669-2 30305116 PMC6180572

[B41] Schmied L. Höglund P. Meinke S. (2021). Platelet-Mediated Protection of Cancer Cells from Immune Surveillance - Possible Implications for Cancer Immunotherapy. Front. Immunol. 12, 640578. 10.3389/fimmu.2021.640578 33777033 PMC7988080

[B42] Siegel R. L. Miller K. D. Fuchs H. E. Jemal A. (2022). Cancer Statistics, 2022. CA A Cancer J. Clin. 72 (1), 7–33. 10.3322/caac.21708 35020204

[B43] Stone R. L. Nick A. M. McNeish I. A. Balkwill F. Han H. D. Bottsford-Miller J. (2012). Paraneoplastic Thrombocytosis in Ovarian Cancer. N. Engl. J. Med. 366 (7), 610–618. 10.1056/NEJMoa1110352 22335738 PMC3296780

[B44] Suvanto M. Beesley J. Blomqvist C. Chenevix-Trench G. Khan S. Nevanlinna H. (2020). SNPs in lncRNA Regions and Breast Cancer Risk. Front. Genet. 11, 550. 10.3389/fgene.2020.00550 32714364 PMC7340126

[B45] Tao D. L. Tassi Yunga S. Williams C. D. McCarty O. J. T. (2021). Aspirin and Antiplatelet Treatments in Cancer. Blood 137 (23), 3201–3211. 10.1182/blood.2019003977 33940597 PMC8351882

[B46] Taucher S. Salat A. Gnant M. Kwasny W. Mlineritsch B. Menzel R. C. (2003). Impact of Pretreatment Thrombocytosis on Survival in Primary Breast Cancer. Thromb. Haemost. 89 (6), 1098–1106. 10.1055/s-0037-1613413 12783124

[B47] Wallace C. H. Wu B. X. Salem M. Ansa-Addo E. A. Metelli A. Sun S. (2018). B Lymphocytes Confer Immune Tolerance *via* Cell Surface GARP-TGF-β Complex. JCI Insight 3 (7). 10.1172/jci.insight.99863 PMC5928869 29618665

[B48] Wurdinger T. In ‘t Veld S. G. J. G. Best M. G. (2020). Platelet RNA as Pan-Tumor Biomarker for Cancer Detection. Cancer Res. 80 (7), 1371–1373. 10.1158/0008-5472.can-19-3684 32075797

[B49] Yang X. Han B. He Z. Zhang Y. Lin K. Su H. (2021). RNA-binding Proteins CLK1 and POP7 as Biomarkers for Diagnosis and Prognosis of Esophageal Squamous Cell Carcinoma. Front. Cell. Dev. Biol. 9, 715027. 10.3389/fcell.2021.715027 34568328 PMC8458940

[B50] Yoshihara K. Shahmoradgoli M. Martínez E. Vegesna R. Kim H. Torres-Garcia W. (2013). Inferring Tumour Purity and Stromal and Immune Cell Admixture from Expression Data. Nat. Commun. 4, 2612. 10.1038/ncomms3612 24113773 PMC3826632

[B51] Zhang B. Wu Q. Li B. Wang D. Wang L. Zhou Y. L. (2020). m6A Regulator-Mediated Methylation Modification Patterns and Tumor Microenvironment Infiltration Characterization in Gastric cancerA Regulator-Mediated Methylation Modification Patterns and Tumor Microenvironment Infiltration Characterization in Gastric Cancer. Mol. Cancer 19 (1), 53. 10.1186/s12943-020-01170-0 32164750 PMC7066851

[B52] Zhang J. Liu J. (2013). Tumor Stroma as Targets for Cancer Therapy. Pharmacol. Ther. 137 (2), 200–215. 10.1016/j.pharmthera.2012.10.003 23064233 PMC3556178

[B53] Zhou C. Wang Y. Wang Y. Lei L. Ji M.-H. Zhou G. (2021). Predicting Lung Adenocarcinoma Prognosis with a Novel Risk Scoring Based on Platelet-Related Gene Expression. Aging 13 (6), 8706–8719. 10.18632/aging.202682 33619234 PMC8034940

[B54] Zhu Q. Meng Y. Li S. Xin J. Du M. Wang M. (2022). Association of Genetic Variants in Autophagy-Lysosome Pathway Genes with Susceptibility and Survival to Prostate Cancer. Gene 808, 145953. 10.1016/j.gene.2021.145953 34500048

