# Supplementary figures and images for: Development of a Platelet-Related Prognostic Model for Colorectal Cancer
Source: Front Genet. 2022 Jun 1;13:904168. doi: 10.3389/fgene.2022.904168 (PMC9198283; doi:10.3389/fgene.2022.904168)

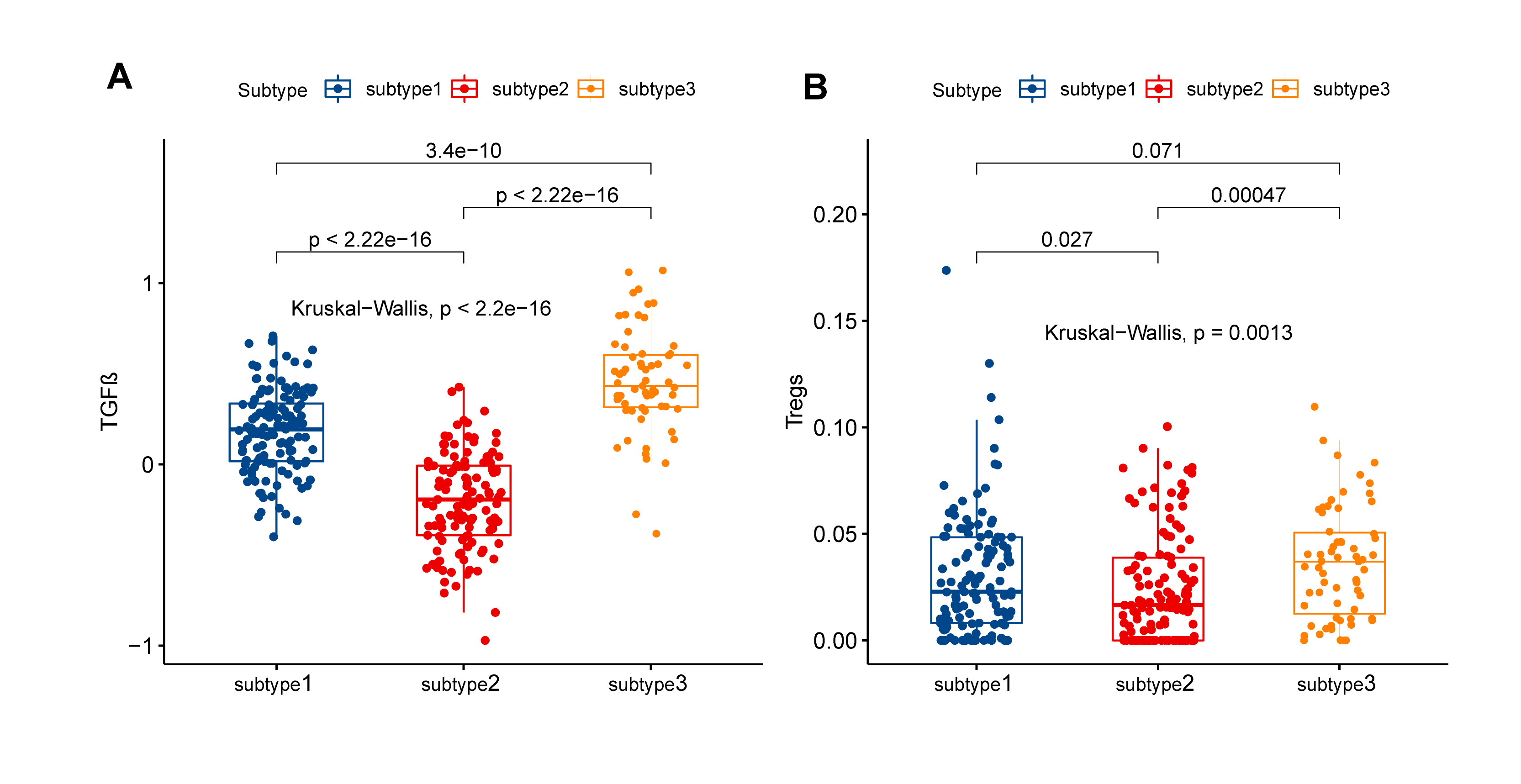

Supplement: Supplementary file 3 [file Image6.TIF]

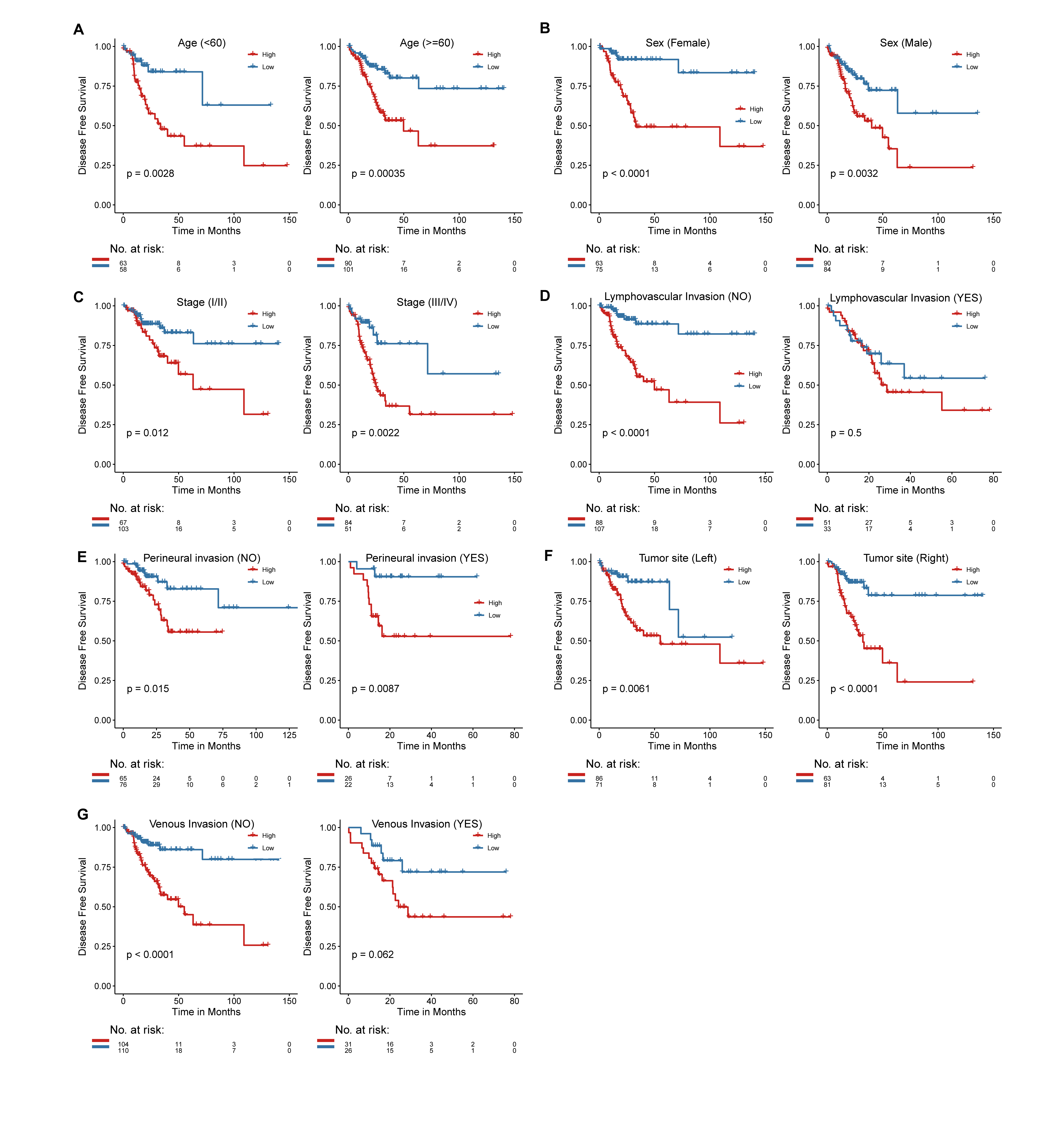

Supplement: Supplementary file 4 [file Image3.TIF]

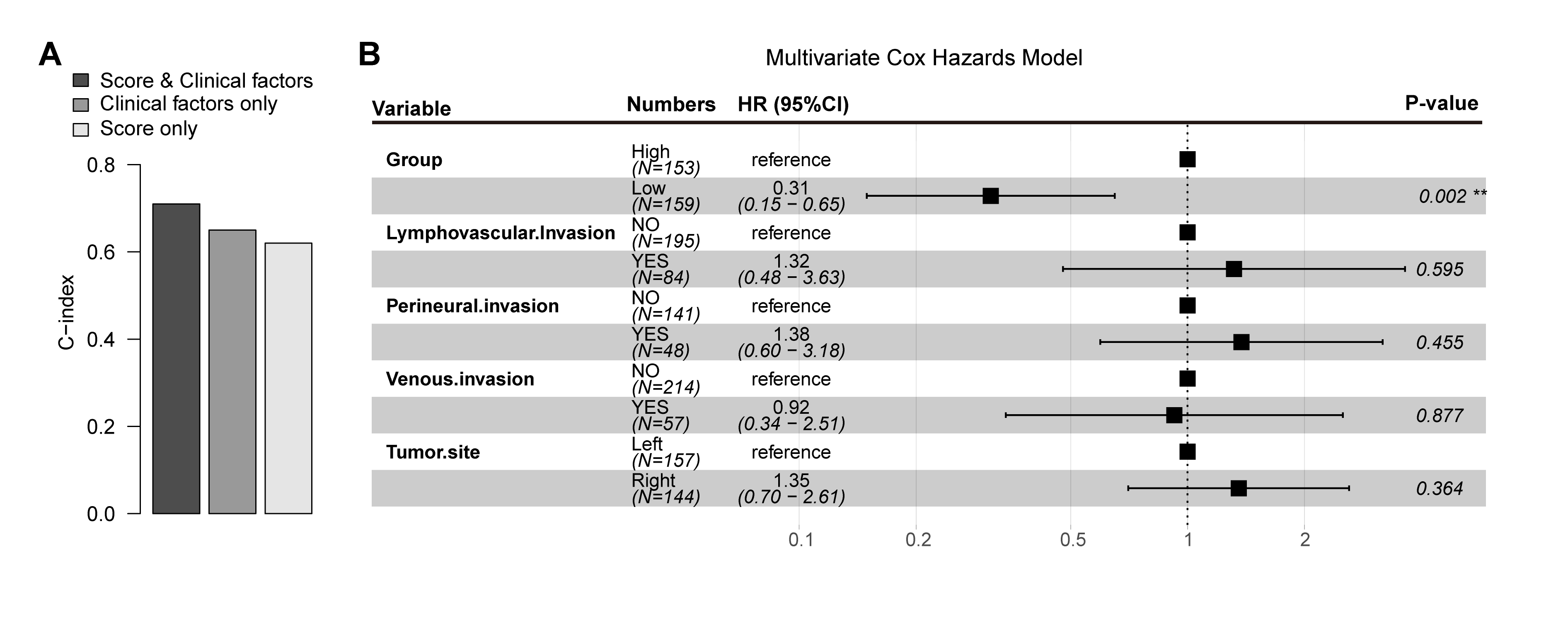

Supplement: Supplementary file 5 [file Image4.TIF]

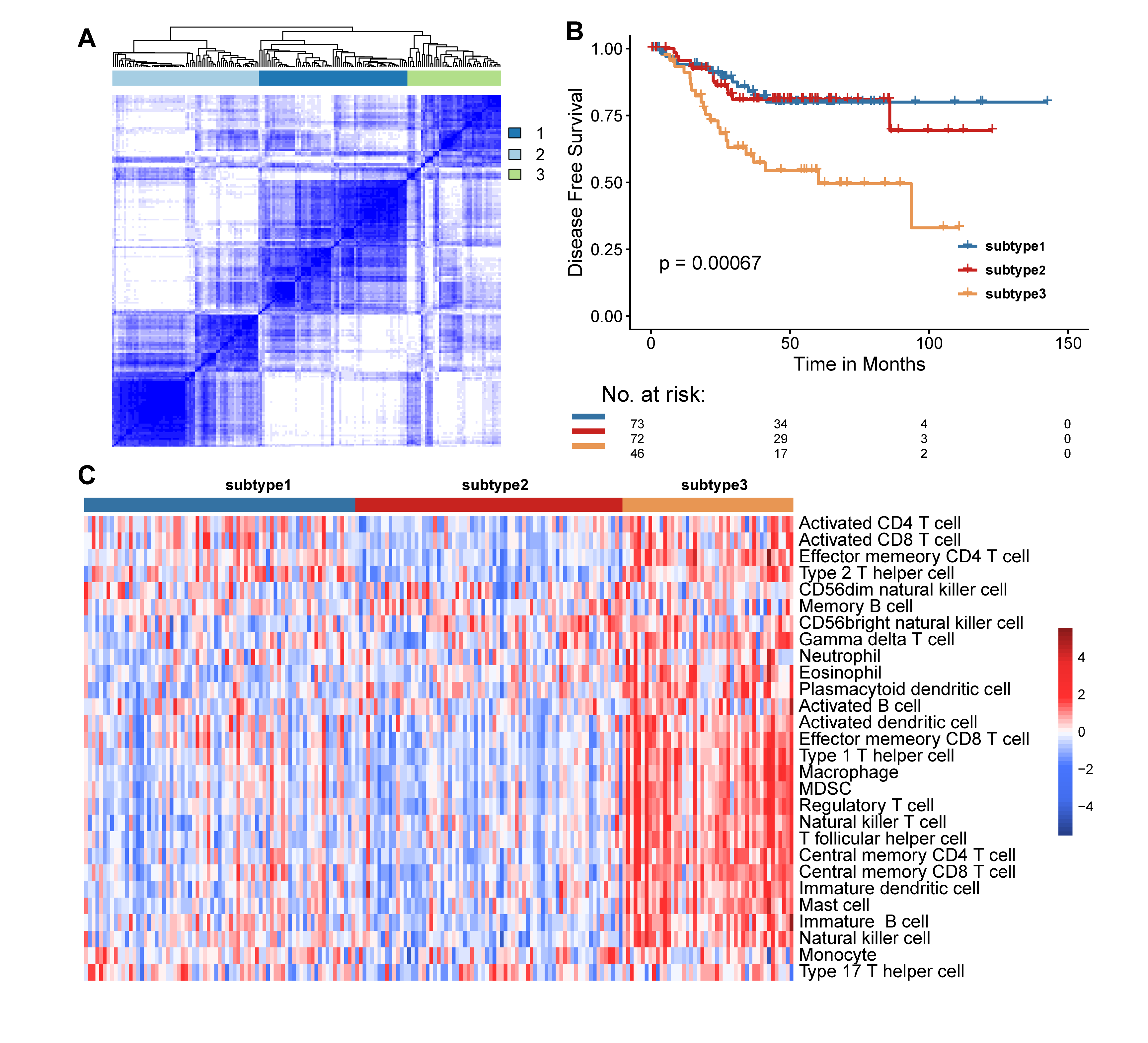

Supplement: Supplementary file 6 [file Image2.TIF]

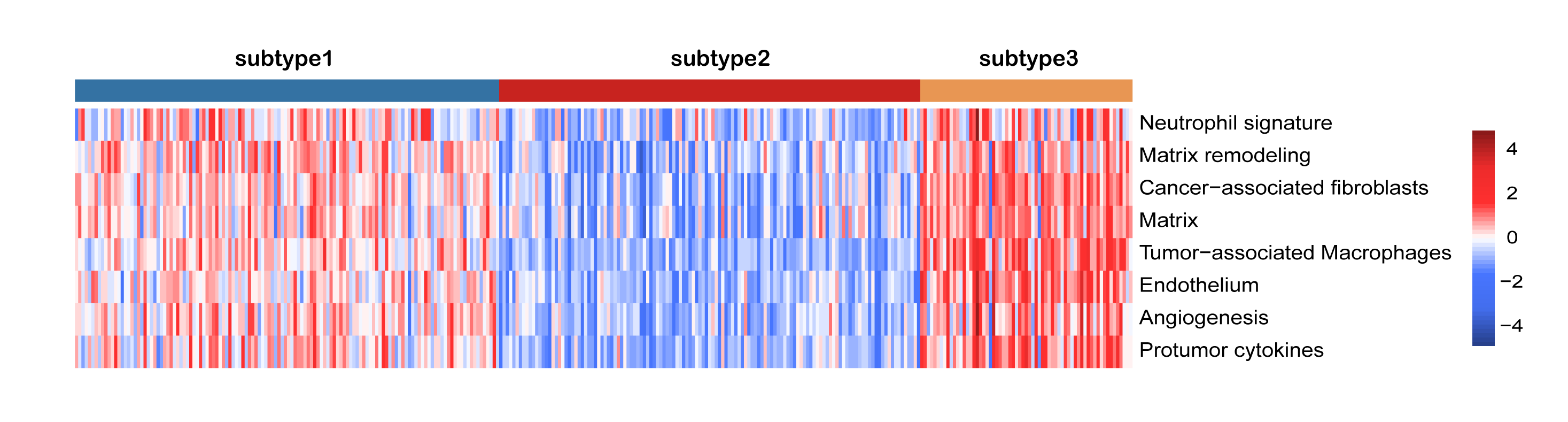

Supplement: Supplementary file 7 [file Image1.TIF]

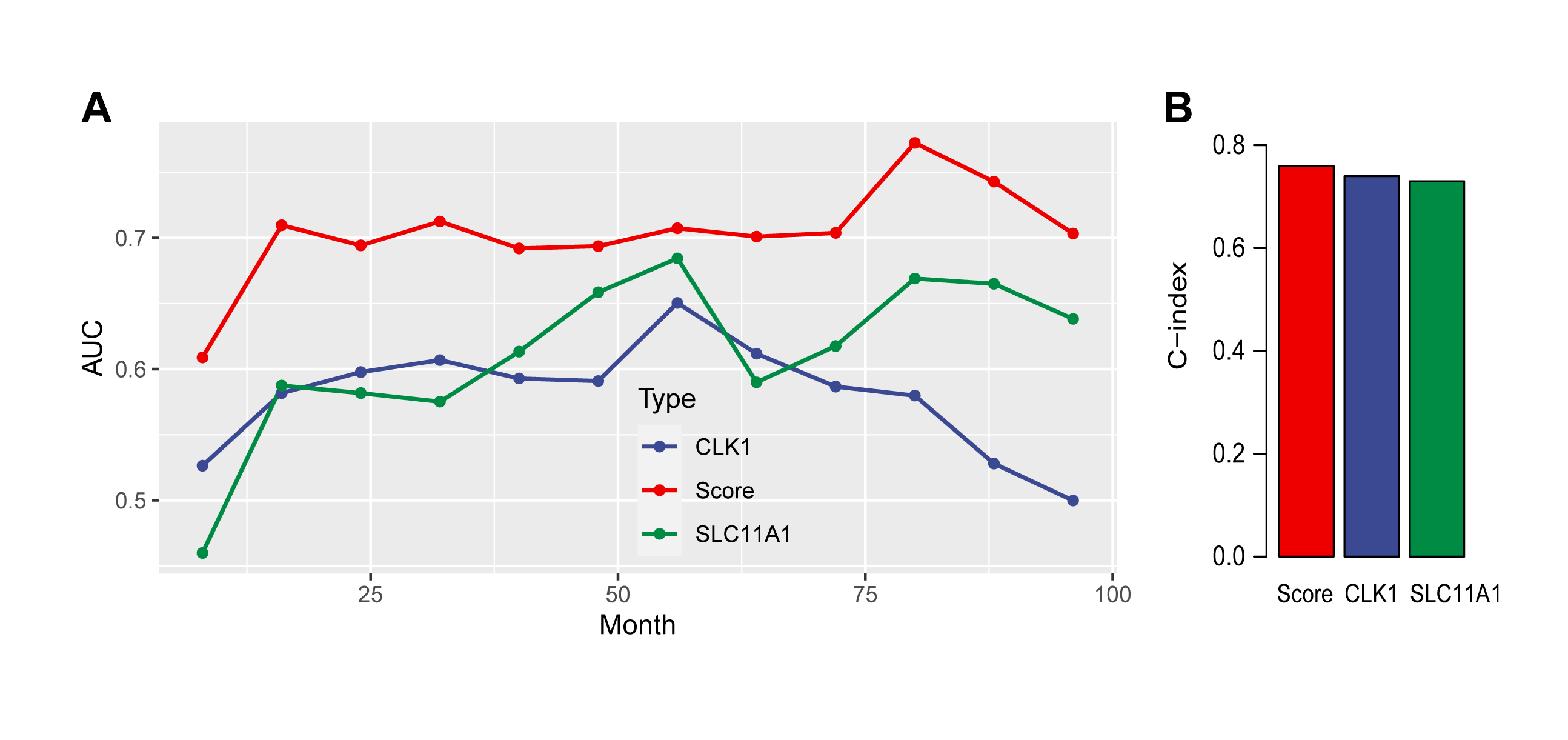

Supplement: Supplementary file 11 [file Image5.TIF]
